# Low-Cost and Environmental-Friendly Route for Synthesizing Nano-Rod Aluminosilicate MAZ Zeolite

**DOI:** 10.3390/molecules27227930

**Published:** 2022-11-16

**Authors:** Fen Zhang, Wei Chen, Lingling Wang, Weiguo Song, Yin Hu

**Affiliations:** 1 Institute of Applied Chemistry, Jiangxi Academy of Sciences, Nanchang 330096, China; 2 Institute of Chemistry, Chinese Academy of Sciences, Beijing 100190, China

**Keywords:** zeolites, low-cost, environmental-friendly, aluminosilicate MAZ, nano-rod

## Abstract

Preparation of nano-rod aluminosilicate Mazzit (MAZ) zeolite under low-cost and environmental-friendly route is attractive, but still challenging. Herein, we report a green route for synthesizing nano-rod MAZ zeolite (MAZ-N) using low-cost and environmental-friendly choline chloride as template. Various characterizations including powder X-ray diffraction (XRD), scanning electron microscope (SEM), N_2_ sorption, and thermogravimetry-differential thermal analysis (TG-DTA) show that MAZ-N samples have good crystallinity and uniform porous structures. Furthermore, the crystallization process and impact of synthesis conditions of MAZ-N samples have been investigated in detail. These results suggest the potential applications of MAZ-N zeolites as supporting catalyst compounds in industrial processes.

## 1. Introduction

As one of the most important microporous materials, zeolites have been widely applied in industrial processes for the production of chemicals and fuels due to their large internal surface area, high hydrothermal stability, unique channel structure, and superior solid acidity [1,2,3,4,5,6,7,8,9,10,11,12]. The chemical composition and pore structure of zeolites are significant, but also the control of their morphology has great effect on their performance in catalytic applications [13,14,15,16,17,18,19,20]. Additionally, the improved mass diffusion ability of nano-rod zeolites would provide plenty of potential opportunities for the further application in catalysis, adsorption and environmental protection.

MAZ zeolite consists of gmelinite cages that are linked in columns parallel to the *c*-axis to build main pores with 12-membered rings [21]. Due to this unique structure, MAZ zeolites show excellent catalytic performance in the methane to methanol [22,23], alkylation of aromatics [24,25], oil cracking [26], hydrocracking [27], and in the isomerization of paraffin and aromatics [28]. There are several MAZ samples that have been successfully synthesized with costly organic template. In the conventional synthesis of zeolite omega, the tetramethylammonium cation (TMA^+^) is used as the organic structure direct agent (OSDA). Both Martins group and Figueras group fabricated MAZ samples using tetramethylammonium hydroxide, which is a classical organic template of MAZ synthesis [29,30]. TMA^+^ may be occluded in the gmelinite cages during omega crystal growth process, which is supposed to be powerful OSDA during the zeolite synthesis [21]. Zones group prepared MAZ zeolites in the presence of dioxane as an organic template [31]. Furthermore, Dong group obtained MAZ zeolites using piperazine at 150 °C for 4 d [32]. However, the above organic templates are expensive, toxic and environmentally unfriendly, which would hinder the further application of MAZ zeolites in petrochemical processes. Thus, the development of a low-cost and environmental-friendly route for synthesizing a nano-rod aluminosilicate MAZ zeolite is still imperative and challenging.

Here, we focus on choline, a low-cost, nontoxic and biodegradable template. Choline is a water-soluble vitamin-like essential nutrient which refers to quaternary ammonium salt containing N,N,N-trimethylethanolammonium cation [33]. Moreover, choline has been successfully applied in synthesizing several zeolite crystals as a structure-directing agent [34,35]. In this study, we reported a green route for synthesizing nano-rod MAZ zeolite using low-cost and environmental-friendly choline chloride as template. The zeolite product obtained had good crystallinity and uniform nano-rod morphology. Moreover, we systematically investigated the crystallization of MAZ zeolite on various synthesis conditions and enhanced the comprehension of crystallization process of the nano-rod such as MAZ zeolite. The combination of low cost and high mass transfer ability presents good opportunities for industrial applications of MAZ zeolite in the future.

## 2. Results and Discussion

**Figure 1:**
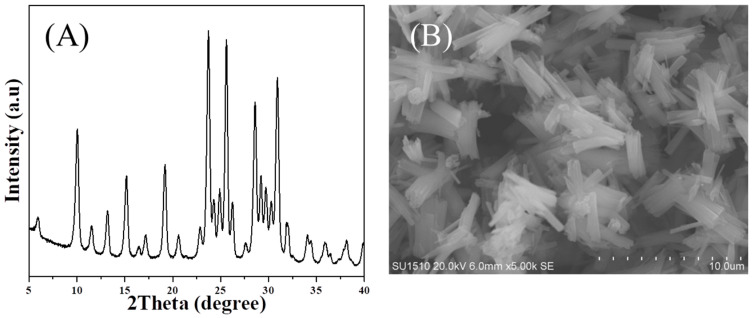
(**A**) XRD pattern and (**B**) SEM image of the as-synthesized MAZ-N sample, respectively.

Figure 1A shows the XRD pattern of the as-synthesized MAZ-N zeolite, which provides a series of characteristic peaks associated with that of the MAZ-C zeolite (Figure S1). These peaks basically remain after calcination, showing good thermal stability of the MAZ-N zeolite. Figure 1B displays the SEM image of the as-synthesized MAZ-N zeolite, showing the uniform assembled nano-rod morphology, which is very different from that of the MAZ-C zeolite (Figure S2). These nanorods are uniform with widths of ca. 200 nm and lengths in the range of ca. 3–5 μm. This result suggests the high quality of MAZ-N zeolite with nano-rod morphology could be successfully obtained, in good agreement with the result from XRD pattern in Figure 1A.

**Figure 2:**
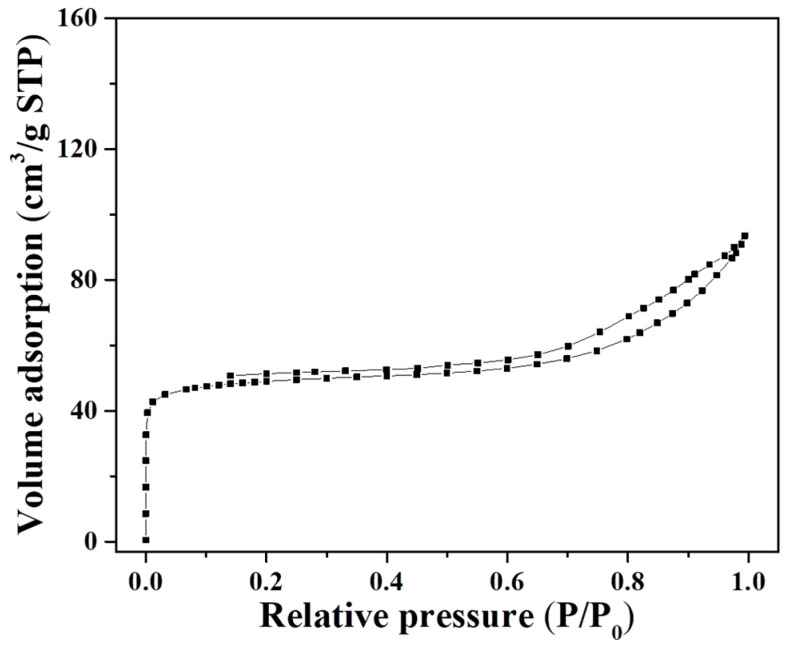
N_2_ sorption isotherm of the H-MAZ-N sample.

Figure 2 shows the N_2_ sorption isotherms of H-MAZ-N zeolite, indicating steep increases in the adsorbed volumes at a relative pressure (10^−6^ < P/P_0_ < 0.01), which is due to the filling of micropores. In addition, the hysteresis loop appeared to be at the relative pressure range of 0.5–0.95. The BET surface area and micropore volume of the MAZ-N zeolite are measured at about 173 m^2^/g and 0.05 cm^3^/g, which are similar to those of the conventional MAZ zeolite reported in the bibliography [36].

**Figure 3:**
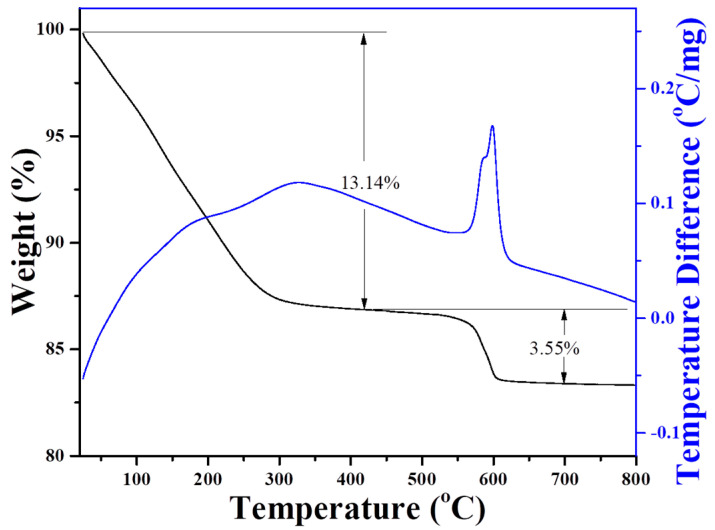
Thermogravimetry–differential thermal analysis (TG-DTA) curves of the as-synthesized MAZ-N sample.

Figure 3 displays the TG-DTA curves of the as-synthesized MAZ-N zeolite in the temperature range from 25 to 800 °C. The weight loss associated with the adsorbed water appears to be below 250 °C, while the weight loss assigned to the combustion of organic template shows that the temperature ranged from 400 to 630 °C. After 700 °C, no obvious weight loss was observed. The DTA curve exhibits a main exothermic peak within the temperature range of 400–630 °C accompanied by the weight loss at approximately 3.55% assigned to the combustion of organic template in the micropores of MAZ-N zeolite. A brief summary of these characterizations confirms that MAZ-N has a good crystallinity and nano-rod morphology. The low-cost and environmental-friendly template could be promising for large batch production of the high-effective MAZ zeolite in industrial processes.

**Figure 4:**
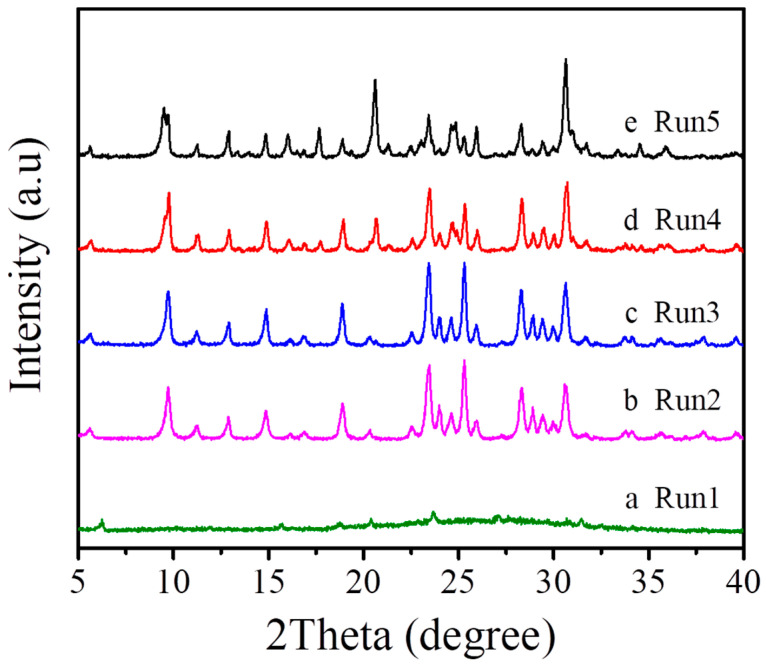
XRD patterns of the samples synthesized with different Na_2_O/SiO_2_ molar ratios ((**a**) Na_2_O/SiO_2_ = 0.187, (**b**) Na_2_O/SiO_2_ = 0.203, (**c**) Na_2_O/SiO_2_ = 0.219, (**d**) Na_2_O/SiO_2_ = 0.234, and (**e**) Na_2_O/SiO_2_ = 0.250).

**Figure 5:**
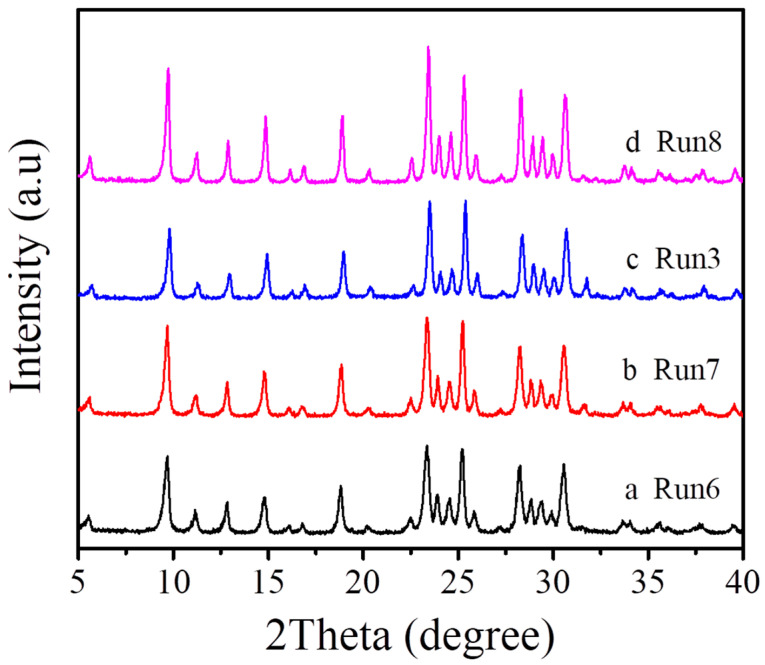
XRD patterns of the samples synthesized with different SiO_2_/Al_2_O_3_ molar ratios ((**a**) SiO_2_/Al_2_O_3_ = 10, (**b**) SiO_2_/Al_2_O_3_ = 12, (**c**) SiO_2_/Al_2_O_3_ = 14, and (**d**) SiO_2_/Al_2_O_3_ = 18).

**Figure 6:**
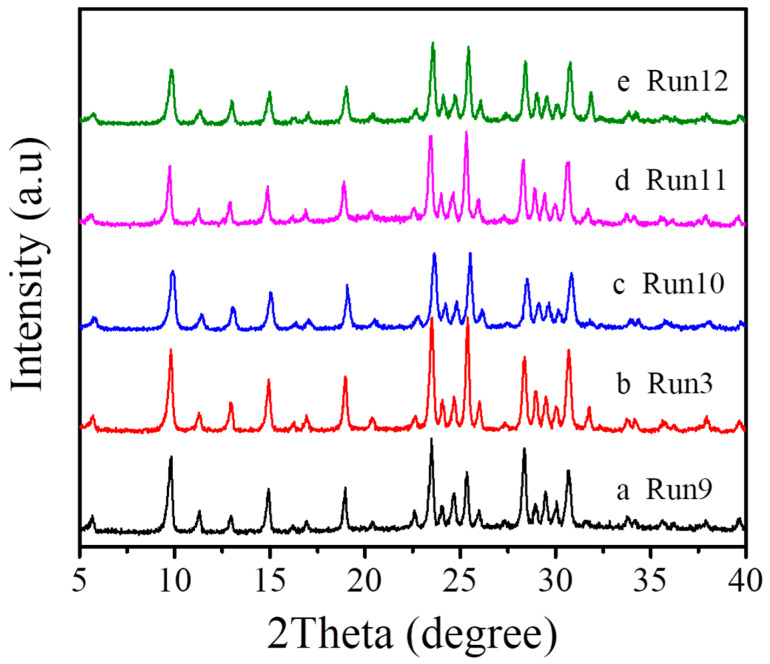
XRD patterns of the samples synthesized with different R/SiO_2_ molar ratios ((**a**) R/SiO_2_ = 0.382, (**b**) R/SiO_2_ = 0.446, (**c**) R/SiO_2_ = 0.511, (**d**) R/SiO_2_ = 0.575, and (**e**) R/SiO_2_ = 0.639).

As shown in Table 1 and Figure 4, Figure 5 and Figure 6, we systematically investigated the crystallization of MAZ zeolite at 150 °C in the presence of low-cost and environmental-friendly choline chloride on various synthesis conditions. When the Na_2_O/SiO_2_ molar ratio in the starting gel is adjusted from 0.187 to 0.250, products with amorphous phases, pure MAZ, a mixture of MAZ with the analcine (ANA) structure are elicited (Run 1–5, Table 1, Figure 4). The Na_2_O/SiO_2_ molar ratio for synthesizing a pure MAZ structure ranges from 0.203 to 0.219 (Run 2–3, Table 1).

Furthermore, when the SiO_2_/Al_2_O_3_ molar ratio in the starting gel is distributed in the range of 10–18, all of the products are pure MAZ zeolites (Run 6–8, Table 1, Figure 5). When the SiO_2_/Al_2_O_3_ molar ratio in the starting gel is higher than 18, the products contain MAZ zeolite and ANA structure.

In addition, the template/SiO_2_ molar ratio in the starting gel is vital and carefully studied. We found that the relatively low template/SiO_2_ molar ratios (below 0.382) in the starting gel would result in a mixture of MAZ with the amorphous phase (Run 9, Table 1, Figure 6). When the template/SiO_2_ molar ratio comes up to 0.446, a pure MAZ zeolite with good crystallinity is obtained (Run 9, Table 1, Figure 6). The relatively high template/SiO_2_ molar ratio has no effect in the crystallization of MAZ zeolite (Run 10–12, Table 1, Figure 6).

After the systematic synthesis, aluminosilicate gel for crystallization of nano-rod MAZ zeolites at 150 °C for 1 day with molar ratio of 3.06 Na_2_O/Al_2_O_3_/14 SiO_2_/6.25 Choline Chloride/164 H_2_O is appropriate.

**Figure 7:**
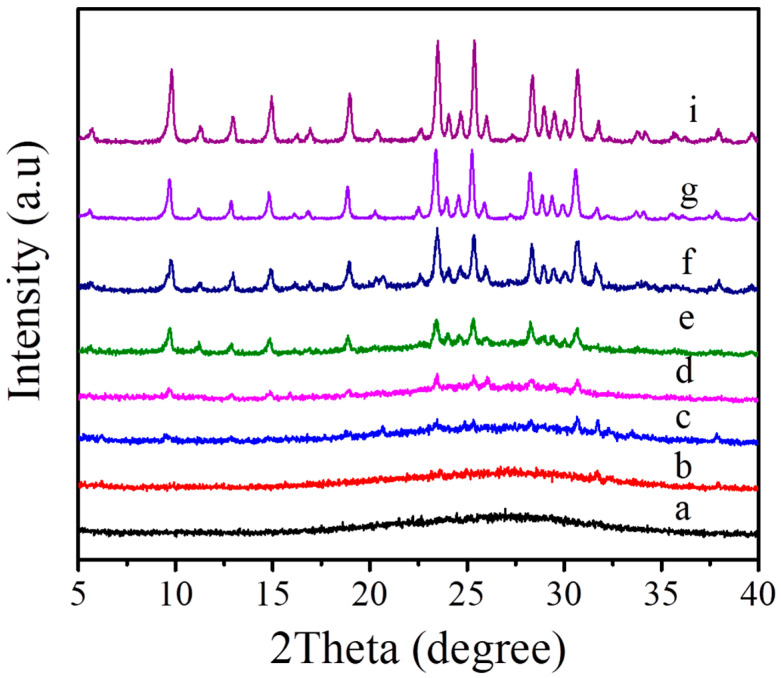
XRD patterns of the as-synthesized MAZ-N samples crystallized at (**a**) 0, (**b**) 12, (**c**) 13.5, (**d**) 15, (**e**) 16.5, (**f**) 18, (**g**) 22.5 and (**i**) 24 h, respectively.

**Figure 8:**
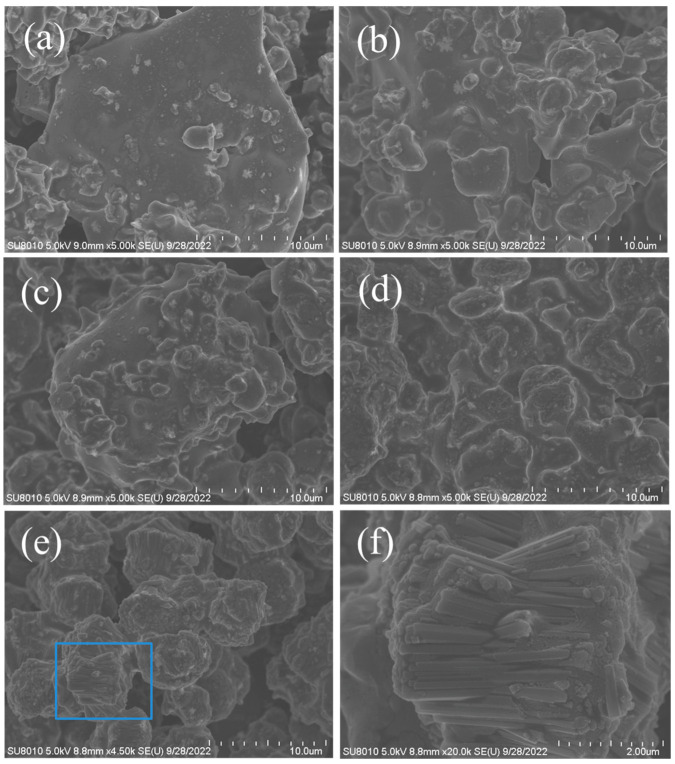
SEM images of the as-synthesized MAZ-N samples crystallized at (**a**) 0, (**b**) 15, (**c**) 16.5, (**d**) 18, (**e**,**f**) 22.5 h, respectively.

**Figure 9:**
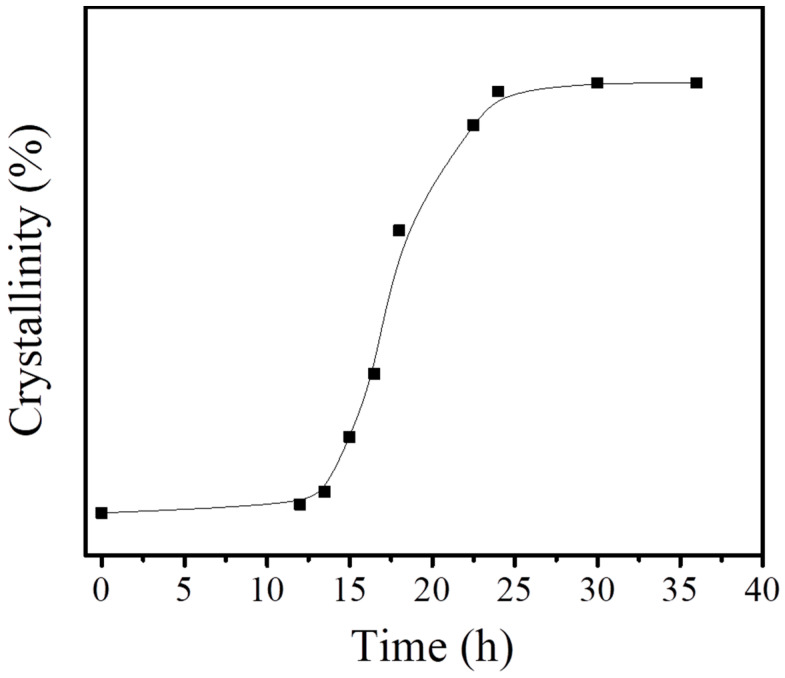
The dependence of the MAZ-N zeolite crystallinity on crystallization time.

Figure 7 displays the crystallization process of the MAZ-N zeolite detected by XRD and SEM techniques. Before crystallization, the XRD pattern shows amorphous phase in the starting aluminosilicate gel (Figure 7a), which is associated with the basically amorphous morphology in the SEM image (Figure 8a). Prolonging the crystallization time from 12 to 15 h causes the peak intensities assigned to MAZ zeolite crystals to become higher (Figure 7b–d). At the same time, several zeolite crystals could be found in the corresponding SEM images (Figure 8b). Further prolonging the crystallization time from 16.5 to 22.5 h causes the peak intensities to continue improving (Figure 7e–g). Correspondingly, much more MAZ zeolite crystals with nano-rod morphology could be observed in the SEM images (Figure 8c–f). When the crystallization time was longer than 24 h, there was no obvious change on peak intensities tested by XRD techniques. In addition, the SEM image (Figure 1B) presents the perfect MAZ zeolite crystals with nano-rod morphology after crystallized for 24 h, suggesting the complete crystallization of the MAZ zeolite. Figure 9 shows the dependence of the MAZ zeolite crystallinity on crystallization time, which is consistent with the *S*-shaped curve of crystal growth.

## 3. Materials and Methods

### 3.1. Materials

The following chemicals were utilized: sodium hydroxide (NaOH, AR, 96%, Sinopharm Chemical Reagent Co., Ltd., Shanghai, China), sodium aluminate (NaAlO_2_, 36.6% Na_2_O, and 43.3% Al_2_O_3_, Sinopharm Chemical Reagent Co., Ltd., Shanghai, China), silica sol (LUDOX HS-40, 40% SiO_2_ in water, Sigma-Aldrich, Shanghai, China), choline chloride (C_5_H_14_ClNO, AR, 98%, Aladdin, Shanghai, China), tetramethylammonium hydroxide (TMAOH, 25% in water, Aladdin, Shanghai, China), and ammonium nitrate (NH_4_NO_3_, AR, 99%, Beijing Chemical Reagent Co., Ltd., Beijing, China) were employed without further purification.

#### 3.1.1. Synthesis of MAZ Zeolite

MAZ zeolite with nano-rod morphology was hydrothermally synthesized at the temperature of 150 °C for 1 day with 3.06 Na_2_O/Al_2_O_3_/14 SiO_2_/6.25 Choline Chloride/164 H_2_O molar ratios of initial synthesis gels in the presence of choline chloride. As a typical run, 0.53 g sodium hydroxide was added to 6.8 g deionized H_2_O, followed by introducing 0.94 g sodium aluminate. After stirring for 1 h, 6.0 g silica sol was added to the mixture dropwise, followed by the last addition of 3.5 g choline chloride when the mixture turned into clear solution. After being stirred at room temperature for 2 h, the final mixture was transformed into an autoclave to crystallize at 150 °C for 1 d. After filtration, wash with deionized H_2_O, air drying, and calcined to remove the organic template, the MAZ zeolite product was collected. The H-form of MAZ sample was prepared by triple ion-exchange with a solution of 1 mol/L NH_4_NO_3_ at room temperature for 2 h and calcination at 500 °C for 4 h, designed as H-MAZ-N zeolite.

#### 3.1.2. Synthesis of Conventional MAZ Zeolite

Conventional MAZ zeolite was hydrothermally synthesized at the temperature of 100 °C for 7 days with 2.4 Na_2_O/Al_2_O_3_/10 SiO_2_/0.24 TMAOH/1100 H_2_O molar ratios of initial synthesis gels according to the previously reported procedure. The product was collected after filtration, washed with deionized H_2_O, air drying and calcined to remove the organic template. The H-form of the sample was prepared by triple ion-exchange with a solution of 1 mol/L NH_4_NO_3_ at room temperature for 2 h and calcination at 500 °C for 4 h, designed as H-MAZ-C zeolite.

### 3.2. Characterization

Powder X-ray diffraction (XRD) (Rigaku, Tokyo, Japan) data were measured at room temperature with a Rigaku Ultimate VI X-ray diffractometer (40 kV, 40 mA) using CuKα1 radiation (λ = 1.5406 Å). Scanning electron microscopy (SEM) (Hitachi, Tokyo, Japan) experiments were acquired with Hitachi SU-1510 and SU-8010 electron microscope. Nitrogen sorption experiments were performed on a Micromeritics ASAP 2460 M (Micromeritics Instrument Corporation, Atlanta, GA, USA) and Tristar system apparatus at −196 °C. The micropore volume and specific surface area were calculated using the t-plot and BET methods, respectively.

## 4. Conclusions

In summary, we developed a low-cost and environmental-friendly route for preparing nano-rod aluminosilicate MAZ zeolite. The obtained MAZ zeolites have advantages of high crystallinity, uniform morphology of a nano-rod, high BET surface area, and good mass transfer ability. Moreover, the crystallization process of MAZ-N zeolites has been investigated in detail, which is consistent with the rule of crystal growth. We hold the opinion that this sustainable green route might offer good opportunities for practical applications of MAZ zeolites as high-efficient catalysts in the future.

## Figures and Tables

**Table 1:** Impact of synthesis conditions on the crystallization of aluminosilicate MAZ-N zeolites (H_2_O/SiO_2_ = 11.7, at 150 °C for 1 d).

Run	Na_2_O/SiO_2_	SiO_2_/Al_2_O_3_	R/SiO_2_ ^a^	Products ^b^	Crystallinity ^c^ (%)
1	0.187	14	0.446	Amor.	/
2	0.203	14	0.446	MAZ	90
3	0.219	14	0.446	MAZ	100
4	0.234	14	0.446	MAZ + ANA	/
5	0.250	14	0.446	MAZ + ANA	/
6	0.219	10	0.446	MAZ	90
7	0.219	12	0.446	MAZ	100
8	0.219	18	0.446	MAZ	125
9	0.219	14	0.382	MAZ + Amor.	/
10	0.219	14	0.511	MAZ	85
11	0.219	14	0.575	MAZ	80
12	0.219	14	0.639	MAZ	76

**^a^** R stands for choline chloride as template. **^b^** The phase appearing first is dominant. **^c^** Run 3 was designated as 100% crystallinity.

## Data Availability

The data presented in this study are available on the request from the corresponding author.

## Supplementary Materials

The following supporting information can be downloaded at: https://www.mdpi.com/article/10.3390/molecules27227930/s1, Figure S1: XRD pattern of the as-synthesized MAZ-C zeolite; Figure S2: SEM image of the as-synthesized MAZ-C zeolite.
