# Supplementary material for: Low-Cost and Environmental-Friendly Route for Synthesizing Nano-Rod Aluminosilicate MAZ Zeolite
Source: Molecules. 2022 Nov 16;27(22):7930. doi: 10.3390/molecules27227930 (PMC9693496; doi:10.3390/molecules27227930)
Supplement: Supplementary file 1 [file molecules-27-07930-s001.zip › molecules-2004476-supplementary.pdf]

Supporting Information

Low-Cost and Environmental-Friendly Route for Synthesizing Nano-Rod Aluminosilicate MAZ Zeolite

Fen Zhang [1], Wei Chen [1], Lingling Wang [1], Weiguo Song [2] and Yin Hu [1,*]

[1] Institute of Applied Chemistry, Jiangxi Academy of Sciences, Nanchang 330096, China
[2] Institute of Chemistry, Chinese Academy of Sciences, Beijing 100190, China
* Correspondence: huyin@jxas.ac.cn

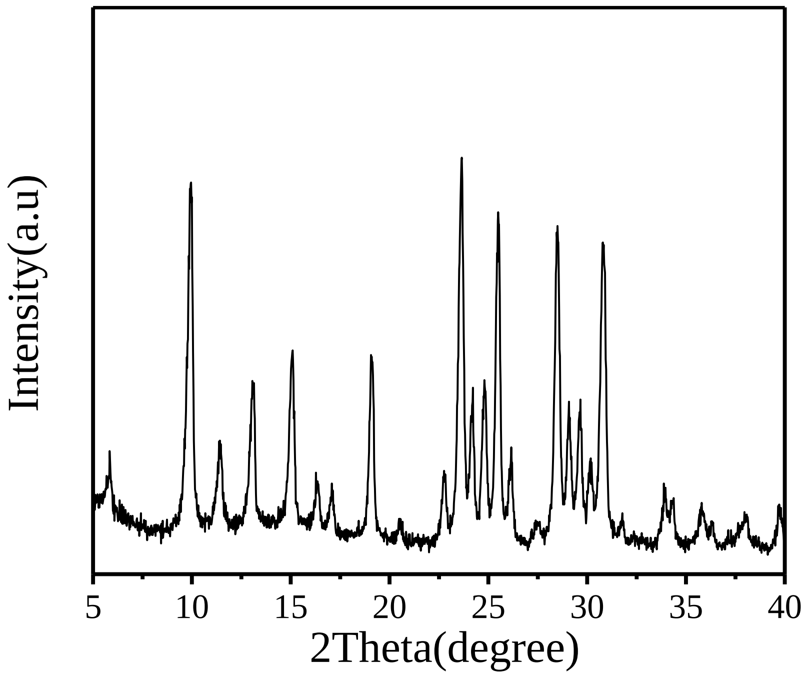

Figure S1. XRD pattern of the as-synthesized MAZ-C zeolite.

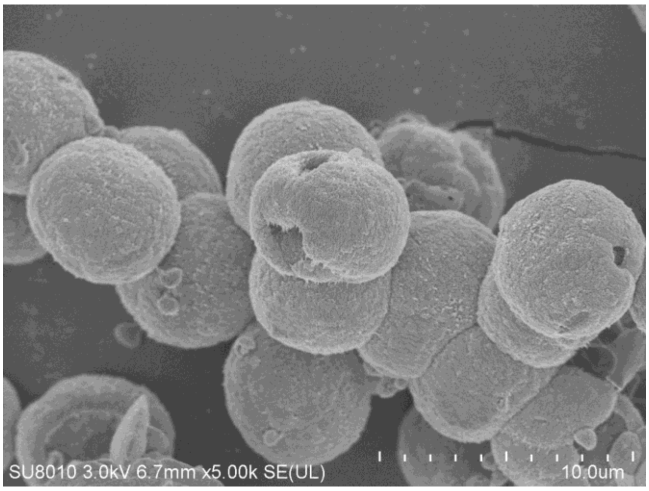

Figure S2. SEM image of the as-synthesized MAZ-C zeolite.
